# Rotavirus Quantification and Genotyping in Wastewater: A Molecular Surveillance Study in Italy (2024–2025)

**DOI:** 10.3390/microorganisms13102319

**Published:** 2025-10-07

**Authors:** Giusy Bonanno Ferraro, Carolina Veneri, Agata Franco, David Brandtner, Daniele Congiu, Pamela Mancini, Marcello Iaconelli, Elisabetta Suffredini, Giuseppina La Rosa

**Affiliations:** 1 National Center for Water Safety (CeNSiA), Istituto Superiore di Sanità, 00161 Rome, Italy; carolina.veneri@iss.it (C.V.); agata.franco@iss.it (A.F.); daniele.congiu@iss.it (D.C.); pamela.mancini@iss.it (P.M.); marcello.iaconelli@iss.it (M.I.); giuseppina.larosa@iss.it (G.L.R.); 2 Department of Infectious Disease, Istituto Superiore di Sanità, 00161 Rome, Italy; david.brandtner@iss.it; 3 Department of Food Safety, Nutrition and Veterinary Public Health, Istituto Superiore di Sanità, 00161 Rome, Italy; elisabetta.suffredini@iss.it

**Keywords:** rotavirus, wastewater-based epidemiology (WBE), quantification, genotyping, urban wastewater, digital RT-PCR

## Abstract

Rotavirus remains a leading cause of acute gastroenteritis worldwide, particularly in young children, despite widespread vaccination efforts. This study aims to evaluate rotavirus circulation at the population level through wastewater-based epidemiology (WBE), offering a non-invasive, complementary approach to clinical surveillance. Between 2024 and 2025, a total of 172 composite 24 h samples were collected from eight urban wastewater treatment plants across Northern, Central, and Southern Italy. Viral RNA was concentrated by PEG precipitation and quantified using digital RT-PCR, while genotypes were determined via nested PCR targeting VP7 and VP4 genes. Rotavirus RNA was detected in 143 out of 172 samples (83.1%), with viral loads ranging between 4.2 × 10^2^ to 7.3 × 10^5^ genome copies per liter (g.c./L). Genotyping revealed G3 as the predominant VP7 type, followed by G1, G2, G6, and G9. All VP4-positive samples were classified as P8. This investigation expands current knowledge of rotavirus epidemiology in Italy using molecular surveillance of urban wastewater. By combining digital RT-PCR and genotyping, it offers a robust framework for integrating WBE into rotavirus monitoring programs, especially in settings where clinical surveillance is limited.

## 1. Introduction

Rotavirus is a major global health challenge, recognized as the leading cause of severe, dehydrating diarrhea and mortality among children under five years of age worldwide. In 2019, rotavirus was responsible for approximately 19% of all diarrhea-related deaths, accounting for over 235,000 fatalities globally. The highest burden is concentrated in low-income regions such as Africa, Oceania, and South Asia, where access to sanitation, nutrition, and healthcare remains limited [1]. Despite significant progress over recent decades, including a marked decline in mortality rates, rotavirus continues to pose a serious threat in vulnerable populations.

Transmission occurs primarily via the fecal-oral route, through contact with contaminated water, food, hands, and surfaces. This mode of transmission enables the virus to persist in environments with inadequate hygiene and sanitation [2]. Rotaviruses belong to the family Reoviridae and are classified into at least nine groups (A–I) based on antigenic properties of the inner capsid protein VP6. Among these, groups A, B, and C infect humans, with group A (RVA) being the most prevalent and clinically significant cause of diarrhoea in children worldwide.

Rotavirus is a double-stranded RNA virus, genotyped based on two outer capsid proteins: VP7 (defining G genotypes) and VP4 (defining P genotypes). To date, 36 G genotypes and 51 P genotypes have been identified. However, six G types (G1, G2, G3, G4, G9, G12) and two P types (P6 and P8) account for approximately 90% of human rotavirus infections globally [3]. Rotavirus evolution is also shaped by reassortment events, including zoonotic transmissions from animal reservoirs, which may introduce novel or rare genotypes into the human population. While rotavirus infections affect all age groups, the highest mortality remains concentrated among young children, although recent data reveal a rising disease burden in elderly populations in some high-income countries [1].

The global introduction of rotavirus vaccines since 2006 [4] has substantially reduced hospitalizations and deaths in many countries. Vaccine coverage and effectiveness, however, vary widely, with efficacy exceeding 90% in high-income regions such as Europe and Australia but falling to lower levels in some low-income countries. These disparities are mostly attributed to differences in healthcare system capacity, nutritional factors, and the diversity of circulating rotavirus strains [5].

The World Health Organization (WHO) recommends the inclusion of rotavirus vaccines in all national immunization programs, emphasizing that the benefits in preventing severe and potentially fatal diarrheal disease far outweigh the very low risks of adverse events. As of 2022, slightly over half of all countries worldwide have incorporated rotavirus vaccines into their immunization schedules [6].

The implementation of the rotavirus vaccination programme in Sicily, which started in 2013, led to a significant reduction in hospital admissions due to rotavirus gastroenteritis, with a documented 47% decrease [7]. At the national level, the proportion of the population receiving vaccinations increased from under 5% during the period 2009–2013 to over 70% by 2019, following the inclusion rotavirus vaccine in the National Immunization Plan [8]. This increase corresponded with a decline in paediatric gastroenteritis hospital admissions from 16.6 to 9.9 per 100,000 inhabitants, thereby preventing approximately 15% of cases. Recent estimates indicate a national uptake of approximately 60% of the population, with higher coverage reported among mothers and urban populations [9].

Rotavirus is not a notifiable disease at the global or European level, resulting in epidemiological data relying largely on localized studies and reported clinical cases rather than comprehensive national surveillance systems. To fill this gap, networks such as EuroRotaNet have been established. EuroRotaNet is a collaborative European initiative that collects molecular and epidemiological data on circulating rotavirus strains across member countries, including Italy. This network aids in characterizing prevalent genotypes and distinguishing wild-type from vaccine-derived strains, thus supporting assessment of vaccination effectiveness. However, EuroRotaNet primarily uses clinical samples and may not fully capture community-wide viral circulation.

Recently, wastewater-based epidemiology (WBE) has emerged as a valuable complementary tool for monitoring enteric viruses, including rotavirus. WBE offers a timely and population-level picture of viral circulation, overcoming limitations inherent in clinical surveillance. The rationale rests on the fact that rotavirus is excreted in high quantities in feces, making sewage an ideal matrix for viral detection and quantification [10].

Multiple international studies have confirmed rotavirus presence in urban wastewater, with a global pooled prevalence of approximately 41% and even higher rates, reaching 68%, in untreated sewage samples. European studies report an even greater prevalence (~56%), underscoring the significant informative potential of this approach for monitoring circulating rotavirus strains in the post-vaccine era [10].

In Italy, however, data on rotavirus detection in wastewater are outdated and limited, with the most recent comprehensive studies dating back to 2010 [11]. This reveals a critical knowledge gap regarding current viral circulation at the national level. To address this gap, our study was specifically designed to provide a current and comprehensive overview of rotavirus circulation in Italy. To provide a geographically representative overview, we selected three Italian regions, Piedmont (North), Lazio (Center), and Sicily (South). Sicily and Piedmont are the two largest regions by surface area, while all three regions rank among the most populous in the country. Their inclusion ensures both territorial and demographic representativeness, enabling meaningful macro-regional comparisons and offering an updated, representative snapshot of rotavirus circulation in Italy

By applying a robust quantitative technique as digital RT-PCR and extensive genotypic analysis across multiple regions and sampling periods, we sought not only to determine the abundance of rotavirus in urban wastewater hence the trend of circulation in the population over time, but also to unravel the genetic diversity of circulating strains and to explore the seasonal and geographical dynamics of viral shedding. This integrated approach ensures a meaningful contribution to both national and international surveillance efforts and supports the ongoing assessment of vaccine impact and viral evolution in the Italian context.

Unlike outdated and mostly qualitative Italian studies, our work provides high-resolution, quantitative, and molecular data on rotavirus circulation that were previously unavailable at the national level, offering a multi-regional and up-to-date molecular overview of rotavirus in Italy to support future surveillance efforts and public health decision-making.

## 2. Materials and Methods

### 2.1. Samples Collection and Processing

From 2024 to 2025, a total of 172 wastewater 24 h composite samples were collected monthly from eight wastewater treatment plants (WWTPs) located in three Italian regions: Piedmont (North), Lazio (Center), and Sicily (South). Figure S1 shows the geographical coverage included in the study. Sample concentration was performed following a standardized national protocol that employs a polyethylene glycol (PEG)-based method for viral concentration [12,13]. Briefly, after inactivation at 56 °C for 30 min, 45 mL of each sample was centrifuged at 4500× *g* for 30 min to remove larger particles and debris. The clarified supernatant (40 mL) was then mixed with 8% PEG 8000 and 0.3 M NaCl, followed by centrifugation at 12,000× *g* for 2 h. After this step, the supernatant was discarded, and the resulting pellet was resuspended in 2 mL of phosphate-buffered saline (PBS, Arlington, VA, USA) for nucleic acid extraction. RNA extraction was conducted using a fully automated magnetic silica-based platform. Subsequently, extracted nucleic acids were purified with the OneStep PCR Inhibitor Removal Kit (Zymo Research, Irvine, CA, USA) to minimize the presence of PCR inhibitors. Purified RNA samples were then stored at −80 °C until further analysis.

### 2.2. Digital PCR

A real-time PCR assay targeting the highly conserved NSP3 gene of rotavirus was adapted for digital PCR (dPCR) on the QIAcuity One 5-plex dPCR system (Qiagen, Hilden, Germany) to enable absolute quantification of viral RNA. Each reaction consisted of a total volume of 40 μL of reaction mix per well, containing 35 μL mix and 5 μL RNA template, using QIAcuity Nanoplate 26k 24-well. Primer and probe concentrations were optimized to 0.8 µM for both forward and reverse primers and 0.4 µM for the probe. Reverse transcription dPCR (RT-dPCR) analyses were carried out using the QIAcuity OneStep Advanced Probe Kit (Qiagen, Hilden, Germany) following standardized protocols. Two technical replicates were run for each sample to ensure accuracy. Amplification conditions consisted of reverse transcription at 50 °C for 30 min, enzyme activation at 95 °C for 2 min, and 45 cycles of denaturation at 95 °C for 15 s and annealing/extension at 60 °C for 30 s. Experiments were conducted in compliance with the Minimum Information for Publication of Quantitative Digital PCR Experiments (MIQE) guidelines [14]. Primer and probes were synthesized by Bio-Fab Research. Details of the primer/probe sequences are provided in Table 1.

Data analysis was performed with the QIAcuity Software Suite version 2.2.0.26, employing a manual global thresholding approach based on positive controls and no-template controls (NTCs). Given the typically low viral concentrations inherent in environmental samples, sample positivity was defined as having at least three positive partitions in a single replicate, or at least two positive partitions in both replicates. The viral concentration (genome copies per microliter of reaction volume, g.c./μL) reported by the instrument was converted to genome copies per liter (g.c./L) of wastewater using the formula:

**Rotavirus** (g.c./L) = [dPCR result (g.c./μ × L) × reaction volume/volume of RNA tested] × 100 × 25

where 100 represents the total volume of extracted RNA (µL) and 25 is the factor accounting for the volume of wastewater processed (40 mL) relative to one liter. Finally, viral loads were normalized by the WWTP flow rate (L/day) and equivalent inhabitant to express the results as genome copies/day/inhabitant.

### 2.3. Statistical Analysis and Plotting

Statistical analyses were conducted using RStudio (v2024.12.1 + 402) using base R functions. Differences between multiple groups were evaluated using the Kruskal–Wallis test, followed by Dunn’s test post hoc tests for pairwise comparisons. Boxplots illustrating log_10_-transformed normalized viral concentrations (genome copies/day/inhabitant), stratified by regions (Lazio, Piedmont, Sicily) were generated with the ggplot2 package (v3.5.2). Statistical significance annotations were added to the plots. To analyze temporal trends in normalized viral load, a generalized additive model (GAM) was fitted using the mgcv package (v1.9.1) in R. The viral load values were log_10_-transformed to stabilize variance. The model included a smooth term for time, implemented as a cubic spline basis, and a categorical variable for region to account for systematic differences between locations. The smoothness parameter was selected automatically via restricted maximum likelihood (REML) as the default package setting, allowing the model to flexibly capture nonlinear trends while avoiding overfitting. Model diagnostics included checks for normality (Shapiro test W = 0.95) and homoscedasticity of residuals, and the effective degrees of freedom for the smoother (6.15). Basis dimension checking indicated that the chosen basis size for the time smoother (k = 9) was adequate, as reflected by a k-index of 0.89 and a *p*-value of 0.13, suggesting adequate flexibility and no evidence of underfitting.

### 2.4. Amplicon Sequencing of G Types and P Types, Phylogenetic Analysis

Characterisation of the G-type was performed by broad-range nested PCR targeting the VP7 gene [16], followed by Sanger sequencing (Eurofins Genomics). In addition, VP4 gene (P-type) characterisation was performed according to the EuroRotaNet protocols, with genotype assignment based on the molecular size of PCR amplicons. Details of the primer sequences are provided in Table 1.

PCR reactions were carried out using 4 μL of RNA template in a total reaction volume of 25 μL with the SuperScript IV One-Step RT-PCR System (Thermo Fisher Scientific, Waltham, MA, USA), and 1 μL of each primer (10 μM). Thermal cycling conditions for VP7 amplification were as follows: 45 °C for 10 min, 98 °C for 2 min; 40 cycles at 98 °C for 10 s, 55 °C for 10 s, and 72 °C for 1 min; final extension at 72 °C for 5 min. For VP4, cycling conditions were the same, but with a 50 °C annealing temperature. Nested PCR was subsequently performed using 2 μL of the initial PCR product under the following conditions: 98 °C for 30 s, 35 cycles at 98 °C for 10 s, 55 °C (VP7) or 45 °C (VP4) for 10 s, 72 °C for 1 min, final extension at 72 °C for 5 min. Standard precautions were implemented to prevent PCR contamination throughout the procedure. PCR amplicons were analyzed by high-sensitivity capillary electrophoresis using the QIAxcel instrument (Qiagen) with the QIAxcel DNA Fast Analysis Kit (50 bp–1.5 kb DNA Size Marker). PCR amplicons were then purified using a Montage PCRm96 microwell filter plate (Millipore, Billerica, MA, USA) prior to sequencing. Purified PCR products underwent Sanger sequencing at Eurofins Genomics.

Genotyping of VP7 partial sequences was performed using the Rotavirus A Genotyping Tool (RIVM; https://mpf.rivm.nl/mpf/typingtool/rotavirusa/ 30 September 2025 which compares query sequences to a curated set of prototype strains. For each sequence, the tool returns the closest genotype assignment, the reference sequence used, the BLAST (BLAST+ 2.17.0) score, and the bootstrap support of the assignment. Phylogenetic relationships were inferred in MEGA X using the Neighbor-Joining method [1] with 1000 bootstrap replicates, and prototype sequences listed in Table S2 were retrieved from GenBank. The optimal tree is shown. Bootstrap percentages (1000 replicates) indicate the proportion of replicate trees in which the associated taxa clustered together. Prototype sequences were those used by the Rotavirus Typing Tool (Table S2) and were downloaded in full from GenBank. To reduce redundancy, identical sequences were collapsed into a single representative prototype, with the number in parentheses indicating the total count of sequences represented. The complete list of identical sequences for each prototype is provided in the Supplementary Materials.

## 3. Results

### 3.1. Viral Loads

**Figure 1:**
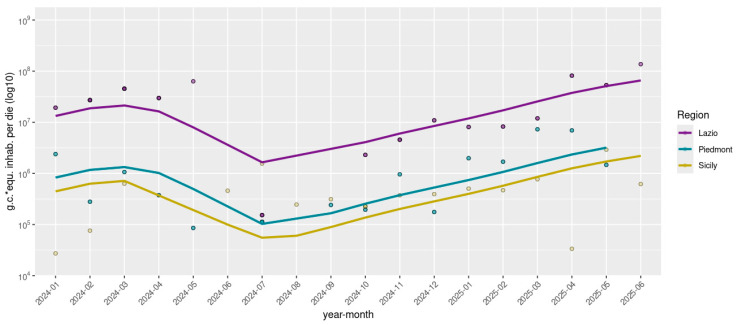
Normalized Rotavirus viral loads detected in eight WWTPs across three Italian regions, grouped by geographical area (North: Piedmont; Center: Lazio; South: Sicily). Viral concentrations are expressed as genome copies (*) per day per inhabitant and are log10-transformed. Samples with viral loads equal to zero were not plotted.

Rotavirus RNA was detected in 143 out of 172 (83.1%) wastewater samples by dPCR, with viral concentrations ranging from 4.2 × 10^2^ to 7.3 × 10^5^ g.c./L. Normalized viral loads (g.c./day × inhabitant) were stratified by regions—Northern Italy (Piedmont), Central Italy (Lazio), and Southern Italy (Sicily), and are represented in Figure 1. Temporal analysis of rotavirus concentrations from early 2024 through mid-2025 revealed distinct regional patterns. Lazio exhibited pronounced fluctuations, with viral loads peaking in early 2024 at 6.3 × 10^7^ g.c./day × inhabitant, followed by a significant decline during the summer months, to 1.5 × 10^5^ g.c./day × inhabitant, and subsequently showing a strong resurgence late in spring 2025, reaching up to 8.2 × 10^7^ g.c./day × inhabitant. Piedmont showed moderate variability with generally lower viral concentrations compared to Lazio, ranging from 8.6 × 10^4^ to 7.3 × 10^6^ g.c./day × inhabitant, characterized by less intense peaks and a noticeable increase in early 2025. In contrast, Sicily displayed a remarkably stable and consistently low level of rotavirus detection, between 3.3 × 10^4^ and 2.9 × 10^6^ g.c./day × inhabitant throughout the entire study period, maintaining relatively constant viral loads from mid-2024 onward. Detailed quantitative data are reported in Table S1.

**Figure 2:**
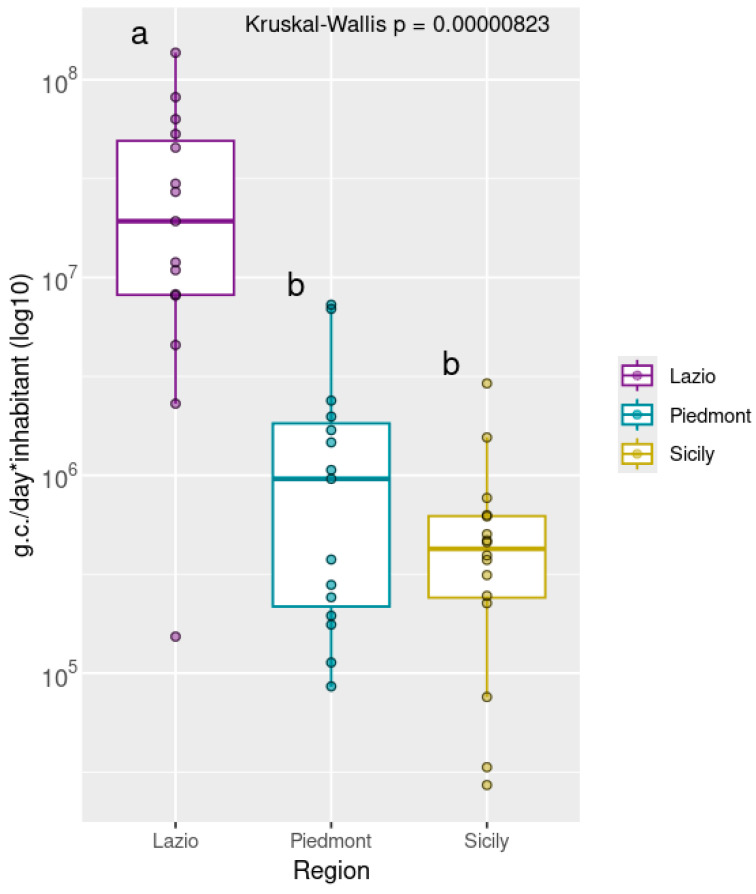
Normalized Rotavirus viral loads, grouped by geographical area North: Piedmont, Center: Lazio, South: Sicily). Viral concentrations are expressed as genome copies per day (*) per inhabitant and are log_10_-transformed. Different letters indicate statistically significant differences between groups (*p* < 0.01). The Lazio region is labeled with “a,” denoting significantly higher viral loads compared to Piedmont and Sicily, which share the letter “b” indicating no significant difference between them. Samples with viral loads equal to zero were excluded from the analysis.

Statistical analysis by Kruskal–Wallis revealed significant differences in viral loads among the three regions (*p* < 0.01). Subsequent Post Hoc comparison using Dunn’s test indicated that the Lazio group had significantly higher viral concentrations compared to both Piedmont and Sicily, which did not differ significantly from each other (Figure 2).

### 3.2. Genotyping

**Figure 3:**
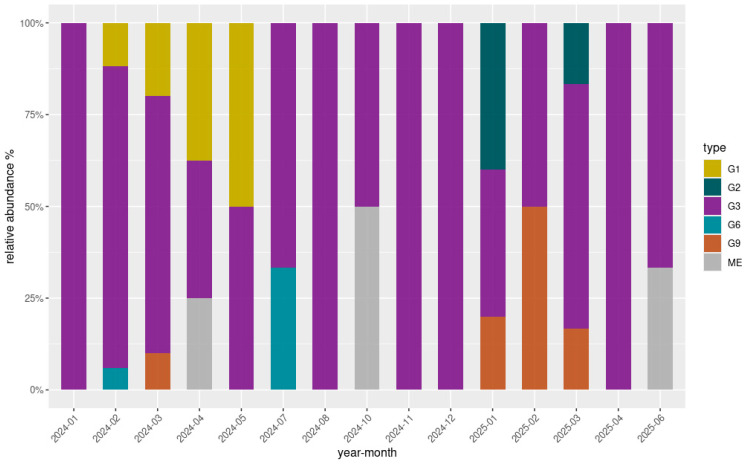
Relative abundance (%) and temporal distribution of genotypes detected in the Lazio region over the study period. The grey segments (labelled “ME”, mixed electropherograms) correspond to months in which VP7 sequences were available but did not yield a clear single genotype, likely due to mixed infections or ambiguous electropherogram signals. These ambiguous results are typical of environmental matrices, where multiple viral strains may co-circulate.

Overall, 80 out of 172 samples (46.5%) exhibited successful amplification of the VP7 gene Via nested PCR, while the amplification of the VP4 gene was achieved in 32 samples (18.6%). Among these, high-quality sequences were obtained for 75/80 (93.8%) VP7 amplicons and 26/32 (81.3%) VP4 amplicons through Sanger sequencing. Ambiguous or low-quality sequencing results, primarily attributable to mixed electropherogram signals indicative of multiple viral genotypes, a common feature in environmental samples, were observed in five VP7 and six VP4 sequences. One additional VP7 sequence was excluded due to incomplete reads. All sequences were successfully genotyped by the Rotavirus A Genotyping Tool, with assignments supported by high BLAST scores and high cluster support values (≥90%). Analysis of the VP7 consensus sequences revealed five G-types circulating within the sampled population: G1 (8/75; 10.7%), G2 (3/75; 4%), G3 (57/75; 76%), G6 (2/75; 2.7%), and G9 (5/75; 6.6%). Notably, all five G-types were detected in the Lazio region only. The G3 genotype was instead present in all three regions, distributed as follows: Lazio (46/57; 80.7%), Piedmont (7/57; 12.3%), Sicily (4/57; 7%), and was the only genotype detected in Piedmont and Sicily. Due to the limited genetic variability observed in Piedmont and Sicily, these regions were not included in genotype diversity illustrations. Figure 3 displays the relative abundance and temporal distribution of the VP7 genotypes in Lazio. Overall, genotype G3 was consistently predominant throughout the study period, representing the majority of detections in most months. However, other genotypes such as G1, G9, and G6 appeared sporadically, particularly during the first half of 2024. G1 showed a marked presence between February and May 2024, while G9 was mainly detected between March and May 2024 and reemerged in early 2025. Genotype G6 was observed only in July 2024, and G2 appeared in January and March 2025. Regarding VP4 genotype characterization, all high-quality sequences (26/32) were all classified as P8 types, indicating a uniform P genotype circulation across the sampled wastewater.

**Figure 4:**
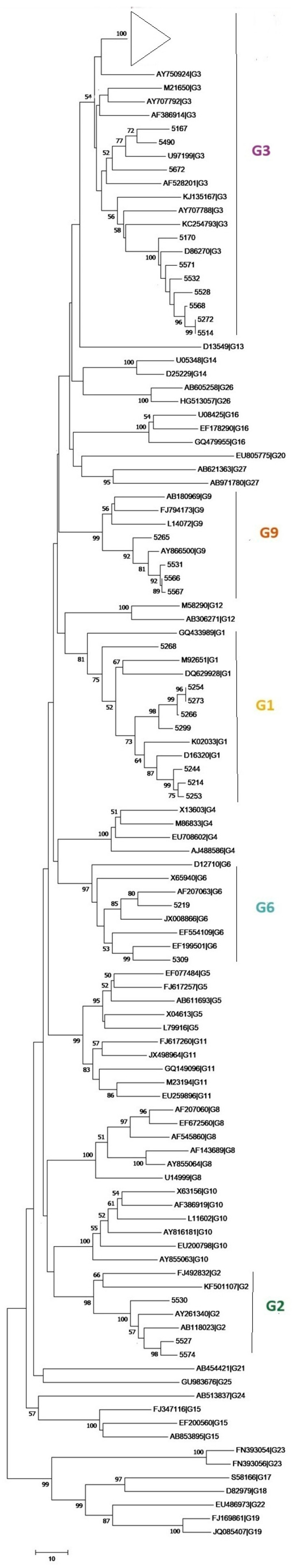
Phylogenetic tree of rotavirus VP7 gene sequences. The evolutionary history was inferred using the Maximum Likelihood method and the General Time Reversible (GTR) model. The tree is drawn to scale, with branch lengths measured in the number of substitutions per site. Bootstrap values above branches indicate statistical support. The triangle represents a cluster of wastewater samples identified as group G3 (refer to Table S1). Evolutionary analyses were conducted in MEGA12.

Figure 4 presents the phylogenetic tree constructed from the VP7 gene sequences obtained in this study, alongside relevant reference strains (Table S2). Sequences for which a consensus could not be generated, due to mixed electropherogram signals or partial sequences, were excluded from the analysis. The phylogenetic tree showed that all major genotypes (G1, G2, G6, G9) clustered as monophyletic groups with high bootstrap support (≥90%). Genotype G3 sequences, however, did not form a single strongly supported cluster despite being assigned to the G3 genotype by the Rotavirus Typing Tool. Instead, they appeared interspersed within multiple closely related branches, reflecting low phylogenetic resolution for this group in the VP7 fragment analysed. All consensus sequences obtained in this study were submitted to GenBank (Table S3).

## 4. Discussion

Rotavirus continues to represent a significant public health concern on a global scale, particularly among paediatric populations, despite the substantial progress achieved through widespread vaccination programmes. In Italy, the prevalence of rotavirus infections remains underrecognised at the national level due to the absence of mandatory notification and fragmented clinical surveillance [20]. Furthermore, while the majority of clinical surveillance systems concentrate on paediatric cases, WBE captures virus shedding across all age groups, including elderly individuals, who have been increasingly recognised as an at-risk population for rotavirus-related morbidity in high-income countries [21]. In this context, wastewater-based epidemiology (WBE) emerges as a powerful, non-invasive approach offering population-level insights into viral circulation that would otherwise remain undetected. O The application of WBE has evolved significantly over the past two decades. Initially employed in the context of poliovirus eradication programmes, this approach has since been established as a method for the monitoring of a wide range of enteric and respiratory viruses at the community level—including norovirus, adenovirus, hepatitis A, and rotavirus [22,23]. The WBE has recently been expanded to include respiratory pathogens, most notably SARS-CoV-2, thereby demonstrating its value during the course of the COVID-19 pandemic as a near-real-time indicator of community-level transmission. More recently, innovative studies have also begun to explore the potential of WBE for monitoring emerging vector-borne viruses [24], highlighting the growing importance of this approach within integrated One Health surveillance frameworks.

One of the pioneering international studies employed molecular techniques, such as RT-PCR, to detect and characterise rotavirus RNA in sewage sludge [25]. This early work established the foundations for subsequent environmental surveillance initiatives by demonstrating the reliability of identifying rotavirus genomes in wastewater, thereby substantiating the viability of WBE for rotavirus research. Similarly, among the first Italian contributions, important molecular characterization of rotavirus strains detected in water environments was provided, marking a crucial step in the establishment of environmental surveillance within the Italian context [26]. Their work not only confirmed rotavirus presence but also highlighted the genetic diversity of circulating strains in wastewater samples. Since these foundational studies, advances in molecular quantification and genotyping methods have expanded the practical applications of WBE, enabling detailed population-level monitoring of rotavirus dynamics as presented in this study.

In this context, our study aims to: (i) quantify rotavirus abundance in urban wastewater across three Italian regions, hence the trend of circulation in the population; (ii) characterize the genetic diversity of circulating strains; and (iii) assess seasonal and spatial trends in viral shedding. These objectives will help to provide comprehensive insight into rotavirus circulation at the national level, addressing current knowledge gaps and supporting the national surveillance framework.

Our study represents one of the first comprehensive molecular surveillance efforts for rotavirus in Italy, integrating digital RT-PCR quantification and genotyping on untreated urban wastewater samples collected longitudinally across three regions, Piedmont, Lazio, and Sicily, representing North, Central, and South Italy, respectively, over an 18-month period. The detection of rotavirus RNA in over 83% of samples confirms the persistent and widespread shedding of the virus in the Italian population. This occurs despite national vaccination coverage having reached around 60%, highlighting ongoing community circulation [9].

Rotavirus RNA was detected in 83.1% of wastewater samples, with concentrations ranging from 4.2 × 10^2^ to 7.3 × 10^5^ g.c./L. These results align with earlier findings reporting Rotavirus presence in 91.4% of sewage samples, with viral concentrations between 2.2 × 10^2^ and 4.1 × 10^5^ g.c./L [27]. Similar intermediate detection rates and seasonal fluctuations have also been observed in Brazil and the United Kingdom, where differences in vaccination coverage and epidemiological factors shape viral dynamics [28,29].

The viral loads measured in this study—ranging from 4.2 × 10^2^ to 7.3 × 10^5^ genome copies per liter (g.c./L)—are in line with values reported in previous environmental surveillance studies across various geographic regions.

Concentrations ranging from 1.2 × 10^2^ to 1.5 × 10^6^ genome copies per liter (g.c./L) were detected in untreated sewage samples collected in Eastern China [30]. Viral presence was also found in 91.4% of wastewater samples from Argentina, with concentrations between 2.2 × 10^2^ and 4.1 × 10^5^ g.c./L [27], while similar viral loads were reported in raw sewage from Brazil [28]. These findings reinforce the interpretation that the concentrations observed in our study reflect sustained viral shedding within the Italian population, despite the widespread introduction of vaccination. The distinct regional patterns we observed in viral loads, when compared with vaccination data, reveal a key insight into the epidemiological dynamics of rotavirus in Italy. According to a recent national study [9], vaccination uptake is quite homogeneous across the country, with rates of 61.9% in Northwestern Italy (Piedmont), 55.9% in Central Italy (Lazio), and 60.9% in Insular Italy (Sicily). Despite this similarity in vaccination coverage, our data show a clear difference in the viral shedding patterns: Lazio exhibited markedly higher viral loads, Piedmont lower concentrations, and Sicily stable and consistently low levels throughout the study period. This finding highlights how environmental factors, host immunity, and other local variables may influence viral circulation even when vaccine uptake is comparable. A limitation of our analysis, however, is that only aggregated estimates of regional vaccination uptake were available, and more detailed and updated data could not be accessed for the study period. Such information would allow a more direct assessment of the relationship between vaccination coverage and viral loads observed in wastewater.

From a methodological standpoint, our use of chip-based digital PCR (dPCR) provided high sensitivity and reproducibility, enabling accurate quantification even in samples with low viral abundance. This approach is consistent with recent applications of droplet digital PCR (ddPCR) (RT-ddPCR) for rotavirus surveillance in wastewater, as evidenced by studies conducted in India [31] and Kenya/USA [32]. The aforementioned studies similarly demonstrated the superior sensitivity of digital PCR in comparison to conventional qPCR, particularly when analysing complex matrices such as untreated sewage. A regional analysis of the data reveals significant variations in viral load and temporal patterns. Lazio consistently exhibited the highest concentrations, with a first peak in January–February 2024, followed by a decline during mid-2024, and a marked resurgence from late 2024 onwards, reaching its highest levels in June 2025. In Piedmont, viral loads remained generally lower than those observed in Lazio, following a U-shaped temporal trend. After a gradual decline from early 2024 through the summer months, concentrations began to rise steadily in late 2024, reaching higher levels in early 2025. Sicily displayed the lowest and most stable levels of rotavirus RNA, with minor fluctuations and a relatively flat trend from mid-2024 onward. These regional disparities likely reflect a complex interplay of factors, including differences in vaccine coverage, demographic distribution, urban density, climatic conditions, and social behaviours such as travel and seasonal activities. Interestingly, the marked North-to-South gradient in viral load contrasts with more homogeneous patterns reported in environmental surveillance from Germany and the Netherlands [29], warranting further investigation into region-specific influences on rotavirus circulation.

The temporal distribution of rotavirus viral loads exhibits the classical epidemiological pattern observed in temperate climates, with increased circulation during the colder months and a decline in summer. The emergence of a spring peak in Lazio, in particular, could be indicative of waning immunity, increased transmission due to social or environmental factors such as school reopening, or travel-related introductions. These patterns reinforce the value of WBE as an early warning and monitoring tool, especially in contexts where clinical data are delayed, patchy, or underreported.

Molecular characterization of rotavirus in our study revealed a predominance of genotype G3 across all regions examined, representing a notable shift from historical surveillance data in Italy, where genotype G1 was predominant prior to vaccine introduction [33]. A clear transition was documented in Sicily, the first Italian region to implement Rotarix^®^ vaccination in 2012. Their long-term epidemiological surveillance demonstrated a significant decline in G1P strains post-vaccination, with the emergence and eventual predominance of equine-like G3P strains during the 2018–2019 season. This genotype shift, accompanied by increased genetic diversity including G2 and G9, aligns well with our findings and mirrors similar international observations in other post-vaccination settings such as Australia and parts of Europe, where G3 and G9 have gained prevalence, likely driven by vaccine-induced selective pressures and evolving rotavirus ecology [33].

The phylogenetic analysis of G3 sequences further confirmed their predominance but also highlighted a weak clustering pattern. While the genotyping tool consistently assigned all sequences to G3, different phylogenetic reconstruction methods failed to generate a single well-supported clade. This likely reflects the limited resolution of the partial VP7 fragment analyzed, which cannot fully capture the high intratype diversity of G3. Similar findings have been reported in previous studies, indicating that this outcome is due to the inherent heterogeneity of G3 combined with the constraints of the VP7 marker, rather than to shortcomings of the genotyping approach.

Additionally, genotype G6 was detected in wastewater samples, a genotype commonly associated with bovine rotaviruses and rarely found in humans. The detection of genotype G6 Rotavirus in wastewater samples is an uncommon occurrence, yet it is supported by previous epidemiological and molecular surveillance in Italy, where a sporadic circulation of human G6 strains was reported over two decades, highlighting their zoonotic origin and potential for animal-to-human transmission through reassortment events [34]. These G6 strains bear close genetic resemblance to bovine rotaviruses, suggesting the occurrence of cross-species viral evolution and sporadic spillovers into the human population. Complementing this, the first identification of rare G6P [6] and G6P [9] Rotavirus strains in Italian children hospitalized with diarrhoea was reported during the 2011 surveillance season [35]. Importantly, although uncommon, the detection of G6 strains highlights the genetic diversity of circulating rotaviruses and their potential zoonotic origins. Given the urban nature of the wastewater catchment areas, direct contamination from bovine sources is unlikely. Therefore, the presence of G6 in sewage likely reflects sporadic human infections with strains of zoonotic origin—possibly resulting from reassortment events—and underlines the value of wastewater surveillance in capturing signals of unusual or emerging genotypes.

Despite consistent genotyping by computational tools, G3 sequences failed to cluster robustly in the ML tree, likely reflecting the intratype genetic heterogeneity and limited phylogenetic signal in the ~320 bp VP7 fragment. This observation is consistent with previous studies reporting substantial diversity within G3 and the emergence of equine-like lineages that complicate tree resolution based on partial VP7 sequences [36,37]. These findings suggest that the low bootstrap support for G3 clades reflects inherent limitations of the data rather than incorrect genotype assignment. Multiple phylogenetic reconstruction methods and substitution models (including NJ, ML with various best-fit models, and analyses excluding third codon positions) were tested to assess the robustness of the G3 clustering. In all cases, G3 sequences failed to form a single, strongly supported clade, with the sole exception of the UPGMA method. However, UPGMA assumes a strict molecular clock and is generally not considered appropriate for inferring phylogenetic relationships in datasets with rate heterogeneity; therefore, these results are not presented as part of the main findings.

The exclusive identification of P8 strains in VP4 genotyping aligns well with the established molecular epidemiology of rotavirus in Europe, where P8 remains the predominant VP4 genotype detected both in clinical and environmental samples. Across European countries, a predominance of P8 has been reported, often in conjunction with various G-types including G1, G3, and G9 [38]. It is noteworthy that the amplification success for VP4 was lower than for VP7. Given that both genes were targeted using the same RNA extracts and protocols, this discrepancy is unlikely to result from PCR inhibition alone. Rather, it may reflect a greater sequence variability in the VP4 gene, which can reduce primer binding efficiency.

While the findings offer valuable insights, several limitations may have influenced the breadth and resolution of the results. From a spatial perspective, sampling was limited to three regions—one per macro-area—which may not fully capture the geographical heterogeneity of rotavirus circulation across the country. Although samples were collected regularly, the intervals were not evenly spaced, potentially limiting the detection of short-term epidemiological fluctuations. Expanding the number of sites and increasing sampling frequency could further strengthen future surveillance efforts.

A limitation of this study is the monthly sampling frequency, which, while appropriate to capture the seasonal dynamics of rotavirus shedding, may not fully resolve short-term fluctuations. Unlike respiratory viruses, rotavirus is continuously excreted in feces at the population level, and major changes in prevalence typically occur over seasonal rather than daily or weekly time scales. For this reason, monthly sampling was considered adequate and logistically feasible. The few non-uniform intervals observed were attributable to external factors such as national holidays or temporary unavailability of sampling at the wastewater treatment facilities. Future surveillance efforts could benefit from higher-frequency and more evenly spaced sampling to further enhance temporal resolution.

Genotyping success was constrained by the complexity and degradation of environmental RNA, preventing the complete identification of G/P genotype combinations in many samples. Moreover, genotyping was performed using Sanger sequencing only, which, unlike next-generation sequencing (NGS), may fail to detect minority variants or mixed infections. Furthermore, the amplified region does not allow discrimination between vaccine-derived and wild-type genotypes. Data normalization relied on estimated wastewater flow and population served, an approach subject to variability. These challenges, inherent to wastewater samples, including RNA degradation, presence of inhibitors, different primers’ affinity, and frequent co-circulation of multiple strains generating mixed electropherogram signals, likely explain the relatively low amplification success, especially for VP4.

Another important limitation of this study is the lack of integration between wastewater viral load data and clinical indicators such as hospitalizations or case notifications. This constraint was mainly due to the absence of a comprehensive national or regional rotavirus surveillance system in Italy. While our temporal patterns are consistent with those reported in the literature, a direct correlation with clinical outcomes was not feasible. Future studies should prioritize the integration of wastewater data with epidemiological indicators, as this step is essential to maximize the public health relevance of WBE for rotavirus.

Finally, the integration of NGS-based genotyping approaches would enable the detection of mixed infections and low-abundance variants, enhancing molecular resolution. Furthermore, coupling environmental data with clinical surveillance indicators and incorporating standardized normalization strategies—such as the use of human fecal biomarkers—would strengthen the interpretability and public health relevance of wastewater-based rotavirus monitoring.

Despite these limitations, our findings underscore the feasibility and value of integrating wastewater-based epidemiology (WBE) into routine rotavirus surveillance in Italy. The use of digital PCR enables sensitive and near real-time quantification of viral RNA, while genotyping provides insights into strain diversity—together offering complementary data that can support public health authorities in tracking transmission dynamics, optimizing vaccination strategies, and informing timely interventions. Specifically, the analysis of wastewater data can contribute to the evaluation of the effectiveness of vaccination programmes. Indeed, the data revealed a distinct viral circulation pattern in regions with similar vaccination coverage. It has been suggested that the surveillance of wastewater may serve to identify deficiencies in vaccination programmes that may not be apparent from clinical data alone. Furthermore, the detection of viral peaks in wastewater (as observed in Lazio and Piedmont) has the potential to function as an alert system in specific geographical areas. In addition, the surveillance of wastewater can support communication campaigns. In instances where surveillance reveals increasing viral circulation, health authorities can launch targeted campaigns to raise awareness about the importance of vaccination and hygiene, especially in regions with low vaccine uptake or endemic circulation patterns. Moreover, the monitoring of emerging genotypes not covered by current vaccines can provide an early warning for the potential emergence of novel strains and support decisions on the need for updated vaccine formulations.

Overall, WBE offers a valuable population-level perspective on rotavirus activity, revealing sustained viral circulation despite immunization programs and highlighting region-specific differences in viral load and genotype distribution. As such, this approach represents a promising addition to national and international rotavirus surveillance frameworks, with the potential to enhance disease control efforts in pediatric and general populations.

## Figures and Tables

**Table 1:** Primer and probe sequences used in this study.

Region Target	Oligo ID	Nucleotide Sequence (5′-3′)	Size (bp)	References
NSP3	NVP3-FDeg	ACC ATC TWC ACR TRA CCC TC	87	[15]
	NVP3-R1	GGT CAC ATA ACG CCC CTA TA		
	NVP3-Probe	FAM-ATG AGC ACA ATG TTA AAA GCT AAC ACT GTC AA-MGB		
VP7	RV1	GTC ACA TCA TAC AAT TCT AAT CTA AG	1059	[16]
	RV2	CTT TAA AAG AGA GAA TTT CCG TCT G		
	RV3	TGT ATG GTA TTG AAT ATA CCA C	346	
	RV4	ACT GAT CCT GTT GGC CAW CC		
VP4	VP4F	TAT GCT CCA GTN AAT TGG	663	[17,18,19]
	VP4R	ATT GCA TTT CTT TCC ATA ATG		
	2T-1 (P4) *	CTA TTG TTA GAG GTT AGA GTC	362	
	3T-1 (P6) *	TGT TGA TTA GTT GGA TTC AA	146	
	1T-1D (P8) *	TCT ACT GGR TTR ACN TGC	224	
	4T-1 (P9) *	TGA GAC ATG CAA TTG GAC	270	
	5T-1 (P10) *	ATC ATA GTT AGT AGT CGG	462	
	P11 (P11) *	GTA AAC ATC CAG AAT GTG	191	

* Reverse primer used in the nested PCR, paired with the forward primer from the first PCR round.

## Data Availability

The original contributions presented in this study are included in the article/Supplementary Materials. Further inquiries can be directed to the corresponding authors.

## Supplementary Materials

The following supporting information can be downloaded at: https://www.mdpi.com/article/10.3390/microorganisms13102319/s1, Figure S1: Geographical regions included in the study: Lazio (WWTP1–WWTP5); Piedmont (WWTP6–WWTP7); Sicily (WWTP8). Coverage: the geographic coverage area of the WWTPs.; Table S1: Samples analysed in this study—quantitative (g.c./L; g.c./day × inhabitant) and genotyping results (VP7; VP4); Table S2: Rotavirus prototypes retrieved from the Rotavirus Genotyping Tool and employed in the construction of the phylogenetic tree; Table S3: Sequences Accession Number NCBI.

## Abbreviations

The following abbreviations are used in this manuscript:

WWTPs	Wastewater Treatment Plants
WBE	Wastewater Base Epidemiology

## References

[1] Du Y., Chen C., Zhang X., Yan D., Jiang D., Liu X., Yang M., Ding C., Lan L., Hecht R. (2022). Global burden and trends of rotavirus infection-associated deaths from 1990 to 2019: An observational trend study. Virol. J..

[2] US Centers for Disease Control and Prevention (CDC) (2024). Clinical Overview of Rotavirus. https://www.cdc.gov/rotavirus/hcp/clinical-overview/index.html#:~:text=Rotavirus%20is%20primarily%20transmitted%20through,with%20contaminated%20surfaces%20or%20objects..

[3] Antoni S., Nakamura T., Cohen A.L., Mwenda J.M., Weldegebriel G., Biey J.N.M., Shaba K., Rey-Benito G., de Oliveira L.H., Oliveira M.T.D.C. (2023). Rotavirus genotypes in children under five years hospitalized with diarrhea in low and middle-income countries: Results from the WHO-coordinated Global Rotavirus Surveillance Network. PLOS Glob. Public Health.

[4] Abou-Nader A.J., Sauer M.A., Steele A.D., Tate J.E., Atherly D., Parashar U.D., Santosham M., Nelson E.A.S. (2018). Global rotavirus vaccine introductions and coverage: 2006–2016. Hum. Vaccines Immunother..

[5] Prunas O., Asare E.O., Sajewski E., Li Y., Pithawala Z., Weinberger D.M., Warren J.L., Armah G.E., Cunliffe N.A., Iturriza-Gómara M. (2025). Global estimates of rotavirus vaccine efficacy and effectiveness: A rapid review and meta-regression analysis. EClinicalMedicine.

[6] (2013). Rotavirus vaccines WHO position paper: January 2013—Recommendations. Vaccine.

[7] Anzà D., Esposito M., Bertolazzi G., Fallucca A., Genovese C., Maniscalco G., Praticò A.D., Scarpaci T., Vitale E., Restivo V. (2025). Determinants of Rotavirus Vaccine Acceptance in an Area of Southern Italy with Low Vaccination Coverage: A Case-Control Study by the Health Belief Model Questionnaire. Vaccines.

[8] Isonne C., Petrone D., Del Manso M., Iera J., Caramia A., Bandini L., Fadda G., Grossi A., Baccolini V., Costantino C. (2023). The Impact of Rotavirus Vaccination on Discharges for Pediatric Gastroenteritis in Italy: An Eleven Year (2009–2019) Nationwide Analysis. Vaccines.

[9] La Fauci G., Soldà G., Di Valerio Z., Salussolia A., Montalti M., Scognamiglio F., Capodici A., Fantini M.P., Larson H.J., Leask J. (2024). Rates and determinants of Rotavirus vaccine uptake among children in Italy: A cross-sectional study within the 2022 OBVIOUS* project. BMC Public Health.

[10] Awere-Duodu A., Donkor E.S. (2024). Rotavirus in Water Environments: A Systematic Review and Meta-Analysis. Environ. Health Insights.

[11] Ruggeri F.M., Bonomo P., Ianiro G., Battistone A., Delogu R., Germinario C., Chironna M., Triassi M., Campagnuolo R., Cicala A. (2015). Rotavirus genotypes in sewage treatment plants and in children hospitalized with acute diarrhea in Italy in 2010 and 2011. Appl. Environ. Microbiol..

[12] Wu F., Xiao A., Zhang J., Moniz K., Endo N., Armas F., Bushman M., Chai P.R., Duvallet C., Erickson T.B. (2021). Wastewater surveillance of SARS-CoV-2 across 40 U.S. states from February to June 2020. Water Res..

[13] La Rosa G., Iaconelli M., Veneri C., Mancini P., Ferraro G.B., Brandtner D., Lucentini L., Bonadonna L., Rossi M., Grigioni M. (2022). The rapid spread of SARS-COV-2 Omicron variant in Italy reflected early through wastewater surveillance. Sci. Total Environ..

[14] Huggett J.F., dMIQEGroup (2020). The Digital MIQE Guidelines Update: Minimum Information for Publication of Quantitative Digital PCR Experiments for 2020. Clin Chem..

[15] Freeman M.M., Kerin T., Hull J., McCaustland K., Gentsch J. (2008). Enhancement of detection and quantification of rotavirus in stool using a modified real-time RT-PCR assay. J. Med. Virol..

[16] Gilgen M., Germann D., Lüthy J., Hübner P. (1997). Three-step isolation method for sensitive detection of enterovirus, rotavirus, hepatitis A virus, and small round structured viruses in water samples. Int. J. Food Microbiol..

[17] Gentsch J.R., Glass R.I., Woods P., Gouvea V., Gorziglia M., Flores J., Das B.K., Bhan M.K. (1992). Identification of group A rotavirus gene 4 types by polymerase chain reaction. J. Clin. Microbiol..

[18] Iturriza-Gómara M., Green J., Brown D.W., Ramsay M., Desselberger U., Gray J.J. (2000). Molecular epidemiology of human group A rotavirus infections in the United Kingdom between 1995 and 1998. J. Clin. Microbiol..

[19] Iturriza-Gómara M., Kang G., Gray J. (2004). Rotavirus genotyping: Keeping up with an evolving population of human rotaviruses. J. Clin. Virol..

[20] Di Martino G., Cedrone F., D’Addezio M., Odio C., Di Giovanni P., Trebbi E., Tognaccini L., Romano F., Staniscia T. (2024). Incidence of Rotavirus-R elated Hospitalizations in an Italian Southern Region from 2015 to 2021. Diseases.

[21] Aliabadi N., Antoni S., Mwenda J.M., Weldegebriel G., Biey J.N.M., Cheikh D., Fahmy K., Teleb N., Ashmony H.A., Ahmed H. (2019). Global impact of rotavirus vaccine introduction on rotavirus hospitalisations among children under 5 years of age, 2008–2016: Findings from the Global Rotavirus Surveillance Network. Lancet. Glob. Health.

[22] Bosch A., Guix S., Sano D., Pintó R.M. (2008). New tools for the study and direct surveillance of viral pathogens in water. Curr. Opin. Biotechnol..

[23] Haramoto E., Kitajima M., Hata A., Torrey J.R., Masago Y., Sano D., Katayama H. (2018). A review on recent progress in detection methods and prevalence of human enteric viruses in water. Water Res..

[24] Mancini P., Veneri C., Bonanno Ferraro G., Franco A., Iaconelli M., Brandtner D., Lucentini L., Venturi G., Mancuso E., Marsili G. (2025). Detection of Dengue virus RNA in Wastewater during a Local Epidemic in Central Italy (August-October 2024). Food Environ. Virol..

[25] Gallimore C.I., Brown D.W., Gouvea V., Iturriza-Gómara M., Gray J.J. (1997). Molecular characterization of rotaviruses in sewage sludge. Appl. Environ. Microbiol..

[26] Ruggeri F.M., Johansen K., Basile G., Kraehenbuhl J., Svensson L. (1998). Antirotavirus Immunoglobulin A Neutralizes Virus In Vitro after Transcytosis through Epithelial Cells and Protects Infant Mice from Diarrhea. J. Virol..

[27] Barril P.A., Fumian T.M., Prez V.E., Gil P.I., Martínez L.C., Giordano M.O., Masachessi G., Isa M.B., Ferreyra L.J., Ré V.E. (2015). Rotavirus seasonality in urban sewage from Argentina: Effect of meteorological variables on the viral load and the genetic diversity. Environ. Res..

[28] Gutierrez M.B., de Assis R.M.S., Andrade J.D.S.R., Fialho A.M., Fumian T.M. (2023). Rotavirus A during the COVID-19 Pandemic in Brazil, 2020-2022: Emergence of G6P[8] Genotype. Viruses.

[29] Chen J., Grow S., Iturriza-Gómara M., Hausdorff W.P., Fix A., Kirkwood C.D. (2022). The Challenges and Opportunities of Next-Generation Rotavirus Vaccines: Summary of an Expert Meeting with Vaccine Developers. Viruses.

[30] Zhou N., Lv D., Wang S., Lin X., Bi Z., Wang H., Wang P., Zhang H., Tao Z., Hou P. (2016). Continuous detection and genetic diversity of human rotavirus A in sewage in eastern China, 2013–2014. Virol. J..

[31] Nema R.K., Singh A.K., Nagar J., Prajapati B., Sikenis M., Singh S., Diwan V., Singh P., Tiwari R., Mishra P.K. (2024). Investigating the Presence of Rotavirus in Wastewater Samples of Bhopal Region, India, by Utilizing Droplet Digital Polymerase Chain Reaction. Cureus.

[32] Kiulia N.M., Gonzalez R., Thompson H., Aw T.G., Rose J.B. (2021). Quantification and Trends of Rotavirus and Enterovirus in Untreated Sewage Using Reverse Transcription Droplet Digital PCR. Food Environ. Virol..

[33] Bonura F., Mangiaracina L., Filizzolo C., Bonura C., Martella V., Ciarlet M., Giammanco G.M., De Grazia S. (2022). Impact of Vaccination on Rotavirus Genotype Diversity: A Nearly Two-Decade-Long Epidemiological Study before and after Rotavirus Vaccine Introduction in Sicily, Italy. Pathogens.

[34] De Grazia S., Martella V., Rotolo V., Bonura F., Matthijnssens J., Bányai K., Ciarlet M., Giammanco G.M. (2011). Molecular characterization of genotype G6 human rotavirus strains detected in Italy from 1986 to 2009. Infect. Genet. Evol..

[35] Ianiro G., Delogu R., Camilloni B., Lorini C., Ruggeri F.M., Fiore L. (2013). Detection of unusual G6 rotavirus strains in Italian children with diarrhoea during the 2011 surveillance season. J. Med. Virol..

[36] Bányai K., Estes M.K., Martella V., Parashar U.D. (2018). Viral gastroenteritis. Lancet.

[37] Carossino M., Vissani M.A., Barrandeguy M.E., Balasuriya U.B.R., Parreño V. (2024). Equine Rotavirus A under the One Health Lens: Potential Impacts on Public Health. Viruses.

[38] Eurorotanet Annual Report 2023. https://www.eurorotanet.com/wp-content/uploads/2025/04/EuroRotaNet_report-2023_20250331_final_v1.0.pdf.

