# Supplementary material for: Rotavirus Quantification and Genotyping in Wastewater: A Molecular Surveillance Study in Italy (2024–2025)
Source: Microorganisms. 2025 Oct 7;13(10):2319. doi: 10.3390/microorganisms13102319 (PMC12565863; doi:10.3390/microorganisms13102319)
Supplement: Supplementary file 1 [file microorganisms-13-02319-s001.zip › microorganisms-3857343-supplementary.pdf]

Figure S1. Geographical regions included in the study: Lazio (WTP1- WTP5); Piedmont (WTP6 – WTP7); Sicily (WTP8). Coverage: the geographic coverage area of the WWTPs.

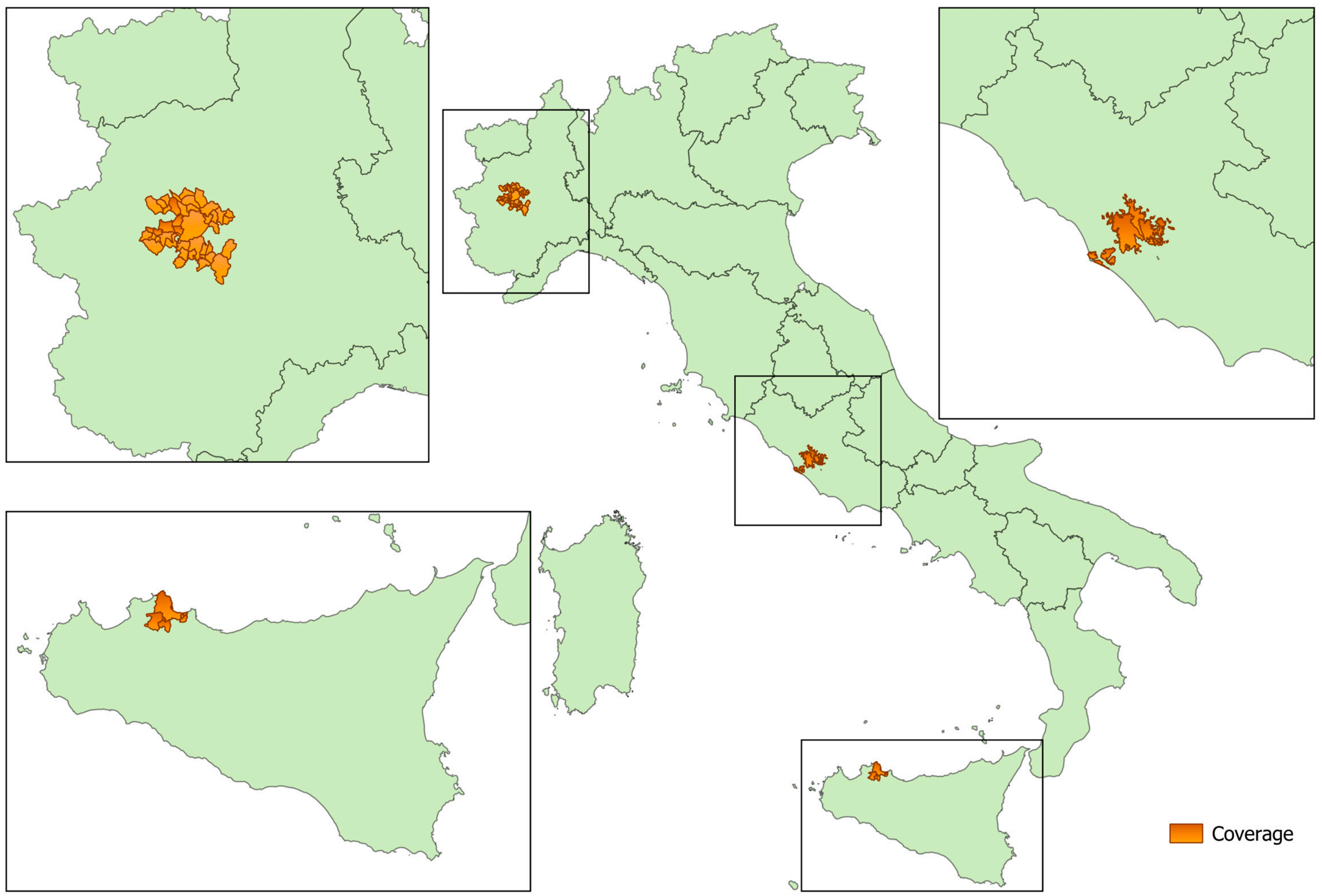

Table S1. Samples analysed in this study – quantitative (g.c./L; g.c./day × inhabitant) and genotyping results (VP7; VP4).

ID sample	Region	WWTPs	Sampling Date	Season	Flow mc/die	Mean conc. [cp/µL] (dPCR reaction)	g.c./L	Population Equivalent	g.c./day × inhabitant	PCR VP7	PCR VP4
5166	Lazio	WWTP1	03/01/2024	Winter	6,48E+05	3,62E+00	7,24E+04	1100000	4,26E+07	G3	P8
5167	Lazio	WWTP2	03/01/2024		1,06E+05	2,25E+00	4,50E+04	300000	1,59E+07	G3	neg
5168	Lazio	WWTP3	03/01/2024		1,72E+05	4,48E+00	8,96E+04	600000	2,56E+07	G3	P8
5206	Piedmont	WWTP6	09/01/2024		2,22E+04	1,63E+00	3,26E+04	180000	4,03E+06	G3	neg
5169	Lazio	WWTP1	10/01/2024		6,39E+05	8,48E-01	1,70E+04	1100000	9,85E+06	G3	P8
5170	Lazio	WWTP2	10/01/2024		9,92E+04	1,59E+00	3,18E+04	300000	1,05E+07	G3	neg
5171	Lazio	WWTP3	10/01/2024		1,03E+05	3,25E+00	6,49E+04	600000	1,11E+07	G3	P8
5210	Piedmont	WWTP7	10/01/2024		4,76E+05	2,15E+00	5,38E+03	3839940	6,66E+05	G3	neg
5259	Sicily	WWTP8	23/01/2024		6,68E+04	1,44E-01	3,59E+02	880000	2,72E+04	neg	neg
5212	Piedmont	WWTP7	24/01/2024		4,50E+05	2,80E+00	7,00E+03	3839940	8,19E+05	G3	P8
5214	Lazio	WWTP2	01/02/2024		1,19E+05	1,62E+00	3,24E+04	300000	1,28E+07	G1	P8
5215	Lazio	WWTP3	01/02/2024		1,67E+05	3,87E+00	7,75E+04	600000	2,16E+07	G3	neg
5216	Lazio	WWTP1	01/02/2024		6,48E+05	2,27E+00	4,53E+04	1100000	2,67E+07	G3	P8
5278	Piedmont	WWTP6	06/02/2024		2,21E+04	1,08E+00	2,69E+03	180000	3,31E+05	neg	neg
5280	Piedmont	WWTP7	07/02/2024		4,60E+05	4,30E-01	1,08E+03	3839940	1,29E+05	neg	neg
5217	Lazio	WWTP2	08/02/2024		1,09E+05	3,70E+00	7,40E+04	300000	2,69E+07	G3	neg
5218	Lazio	WWTP3	08/02/2024		1,44E+05	3,26E+00	6,52E+04	600000	1,56E+07	G3	P8
5219	Lazio	WWTP1	08/02/2024		5,53E+05	4,02E+00	8,05E+04	1100000	4,05E+07	G6	ME
5260	Sicily	WWTP8	13/02/2024		6,98E+04	4,78E-02	9,56E+02	880000	7,59E+04	neg	neg

5241	Lazio	WWTP1	14/02/2024	6,48E+05	5,29E+00	1,06E+05	1100000	6,23E+07	neg	neg
5242	Lazio	WWTP2	14/02/2024	1,06E+05	4,14E+00	8,27E+04	300000	2,93E+07	G3	ME
5243	Lazio	WWTP3	14/02/2024	1,52E+05	4,08E+00	8,16E+04	600000	2,07E+07	G3	P8
5274	Lazio	WWTP4	17/02/2024	7,10E+05	5,28E-01	1,06E+04	780000	9,62E+06	G3	neg
5282	Piedmont	WWTP6	20/02/2024	2,64E+04	9,56E-01	2,39E+03	180000	3,50E+05	neg	neg
5244	Lazio	WWTP1	21/02/2024	2,78E+05	6,00E+00	1,20E+05	1100000	3,04E+07	G1	ME
5245	Lazio	WWTP2	21/02/2024	6,74E+05	4,62E+00	9,24E+04	300000	2,08E+08	G3	neg
5246	Lazio	WWTP3	21/02/2024	1,08E+05	6,57E+00	1,31E+05	600000	2,37E+07	G3	P8
5284	Piedmont	WWTP7	21/02/2024	4,80E+05	9,84E-01	2,46E+03	3839940	3,07E+05	G3	P8
5247	Lazio	WWTP2	28/02/2024	1,60E+05	4,47E+00	8,93E+04	300000	4,76E+07	G3	P8
5248	Lazio	WWTP3	28/02/2024	1,08E+05	7,79E+00	1,56E+05	600000	2,79E+07	G3	P8
5249	Lazio	WWTP1	28/02/2024	1,56E+05	3,35E+00	6,70E+04	1100000	9,50E+06	G3	P8
5496	Lazio	WWTP5	28/02/2024	1,09E+05	8,61E-01	1,72E+04	350000	5,37E+06	G3	neg
5286	Piedmont	WWTP6	05/03/2024	4,77E+04	0,00E+00	0,00E+00	180000	0,00E+00	neg	neg
5250	Lazio	WWTP2	06/03/2024	7,78E+05	2,76E+00	5,52E+04	300000	1,43E+08	G3	P8
5251	Lazio	WWTP3	06/03/2024	7,77E+04	4,53E+00	9,06E+04	600000	1,17E+07	G3	P8
5252	Lazio	WWTP1	06/03/2024	1,09E+05	2,17E+00	4,34E+04	1100000	4,30E+06	G3	P8
5288	Piedmont	WWTP7	06/03/2024	6,42E+05	1,68E-01	4,19E+02	3839940	7,01E+04	neg	neg
5253	Lazio	WWTP2	13/03/2024	1,59E+05	5,42E+00	1,08E+05	300000	5,75E+07	G1	P8
5254	Lazio	WWTP3	13/03/2024	7,08E+05	2,61E+00	5,23E+04	600000	6,17E+07	G1	ME
5255	Lazio	WWTP1	13/03/2024	1,09E+05	5,32E-01	1,06E+04	1100000	1,06E+06	G3	P8
5261	Sicily	WWTP8	18/03/2024	6,44E+04	4,30E-01	8,60E+03	880000	6,29E+05	neg	neg
5498	Lazio	WWTP5	18/03/2024	1,67E+05	7,26E-01	1,45E+04	350000	6,94E+06	neg	neg
5290	Piedmont	WWTP6	19/03/2024	2,66E+04	1,39E+00	2,78E+04	180000	4,12E+06	G3	ME
5256	Lazio	WWTP1	20/03/2024	1,63E+05	5,50E+00	1,10E+05	1100000	1,62E+07	G3	neg
5264	Lazio	WWTP2	20/03/2024	7,17E+05	7,28E+00	1,46E+05	300000	3,48E+08	G3	P8
5265	Lazio	WWTP3	20/03/2024	6,80E+04	4,50E+00	9,00E+04	600000	1,02E+07	G9	P8

5275	Lazio	WWTP4	27/03/2024	Spring	7,10E+05	1,07E-01	2,14E+03	780000	1,95E+06	neg	neg
5294	Piedmont	WWTP6	09/04/2024		3,35E+04	2,39E-01	4,78E+03	180000	8,90E+05	neg	neg
5296	Piedmont	WWTP7	10/04/2024		6,13E+05	1,91E-01	3,82E+03	3839940	6,10E+05	neg	neg
5266	Lazio	WWTP2	15/04/2024		7,08E+05	3,89E+00	7,78E+04	300000	1,84E+08	G1	P8
5267	Lazio	WWTP2	15/04/2024		1,14E+05	2,95E-01	5,90E+03	300000	2,24E+06	G3	neg
5268	Lazio	WWTP3	15/04/2024		1,81E+05	5,88E+00	1,18E+05	600000	3,56E+07	G1	neg
5269	Lazio	WWTP3	15/04/2024		2,75E+05	1,33E-01	2,66E+03	600000	1,22E+06	neg	ME
5270	Lazio	WWTP1	15/04/2024		1,00E+05	4,49E+00	8,98E+04	1100000	8,16E+06	ME	P8
5271	Lazio	WWTP1	15/04/2024		1,00E+05	1,36E+00	2,72E+04	1100000	2,47E+06	ME	neg
5272	Lazio	WWTP4	15/04/2024		1,18E+05	1,67E-01	3,33E+03	780000	5,02E+05	G3	neg
5273	Lazio	WWTP4	15/04/2024		1,18E+05	2,07E+00	4,14E+04	780000	6,24E+06	G1	neg
5316	Lazio	WWTP4	15/04/2024		1,00E+05	4,29E-01	8,59E+03	780000	1,10E+06	G3	neg
5633	Piedmont	WWTP6	23/04/2024		2,74E+04	0,00E+00	0,00E+00	180000	0,00E+00	neg	neg
5634	Piedmont	WWTP7	24/04/2024		5,52E+05	0,00E+00	0,00E+00	3839940	0,00E+00	neg	neg
5635	Piedmont	WWTP6	07/05/2024		4,28E+04	0,00E+00	0,00E+00	180000	0,00E+00	neg	neg
5636	Piedmont	WWTP7	08/05/2024		6,04E+05	0,00E+00	0,00E+00	3839940	0,00E+00	neg	neg
5637	Piedmont	WWTP6	21/05/2024		2,57E+04	1,20E-01	2,40E+03	180000	3,43E+05	neg	neg
5638	Piedmont	WWTP7	22/05/2024		6,00E+05	0,00E+00	0,00E+00	3839940	0,00E+00	neg	neg
5317	Lazio	WWTP4	28/05/2024		1,18E+05	1,59E-01	3,18E+03	780000	4,80E+05	neg	neg
5298	Lazio	WWTP2	29/05/2024		2,64E+05	3,25E+00	6,49E+04	300000	5,70E+07	G3	neg
5299	Lazio	WWTP2	29/05/2024		2,64E+05	1,11E+01	2,22E+05	300000	1,95E+08	G1	neg
5300	Sicily	WWTP8	03/06/2024		6,01E+04	3,36E-01	6,72E+03	880000	4,59E+05	neg	neg
5639	Piedmont	WWTP6	04/06/2024		3,27E+04	0,00E+00	0,00E+00	180000	0,00E+00	neg	neg
5640	Piedmont	WWTP7	05/06/2024		5,99E+05	0,00E+00	0,00E+00	3839940	0,00E+00	neg	neg
5642	Piedmont	WWTP7	19/06/2024		5,32E+05	0,00E+00	0,00E+00	3839940	0,00E+00	neg	neg
5303	Lazio	WWTP2	02/07/2024	Summer	2,49E+05	0,00E+00	0,00E+00	300000	0,00E+00	neg	neg
5306	Lazio	WWTP3	02/07/2024		2,51E+05	0,00E+00	0,00E+00	600000	0,00E+00	G3	neg

5309	Lazio	WWTP4	02/07/2024		1,04E+05	0,00E+00	0,00E+00	780000	0,00E+00	G6	neg
5312	Lazio	WWTP1	02/07/2024		1,60E+05	2,10E-01	4,20E+03	1100000	6,12E+05	G3	P8
5644	Piedmont	WWTP6	02/07/2024		3,83E+04	0,00E+00	0,00E+00	180000	0,00E+00	neg	neg
5645	Piedmont	WWTP7	03/07/2024		5,67E+05	0,00E+00	0,00E+00	3839940	0,00E+00	neg	neg
5318	Sicily	WWTP8	16/07/2024		5,83E+04	1,17E+00	2,34E+04	880000	1,55E+06	neg	neg
5321	Piedmont	WWTP7	23/07/2024		5,44E+05	1,20E-01	2,39E+03	3839940	3,39E+05	neg	neg
5443	Piedmont	WWTP6	03/08/2024		2,77E+04	0,00E+00	0,00E+00	180000	0,00E+00	neg	neg
5467	Sicily	WWTP8	06/08/2024		5,65E+04	1,91E-01	3,83E+03	880000	2,46E+05	G3	neg
5451	Piedmont	WWTP7	04/09/2024		6,45E+05	0,00E+00	0,00E+00	3839940	0,00E+00	neg	neg
5445	Piedmont	WWTP6	18/09/2024		3,63E+04	1,20E-01	2,39E+03	180000	4,83E+05	neg	neg
5468	Sicily	WWTP8	24/09/2024	Fall	5,76E+04	2,39E-01	4,78E+03	880000	3,13E+05	neg	neg
5447	Piedmont	WWTP6	01/10/2024		2,43E+04	0,00E+00	0,00E+00	180000	0,00E+00	neg	neg
5373	Lazio	WWTP2	02/10/2024		2,51E+05	1,62E-01	3,24E+03	300000	2,71E+06	neg	neg
5375	Lazio	WWTP3	02/10/2024		7,10E+05	0,00E+00	0,00E+00	600000	0,00E+00	neg	neg
5377	Lazio	WWTP1	02/10/2024		1,04E+05	2,78E-02	5,56E+02	1100000	5,27E+04	neg	neg
5453	Piedmont	WWTP7	02/10/2024		5,41E+05	0,00E+00	0,00E+00	3839940	0,00E+00	neg	neg
5438	Lazio	WWTP2	09/10/2024		1,66E+05	1,08E-01	2,15E+03	300000	1,19E+06	neg	P8
5439	Lazio	WWTP3	09/10/2024		7,13E+05	1,32E-01	2,64E+03	600000	3,14E+06	neg	neg
5440	Lazio	WWTP1	09/10/2024		1,11E+05	8,11E-02	1,62E+03	1100000	1,64E+05	neg	neg
5463	Sicily	WWTP8	14/10/2024		5,87E+04	1,92E-01	3,83E+03	880000	2,56E+05	G3	neg
5544	Piedmont	WWTP6	15/10/2024		2,49E+04	0,00E+00	0,00E+00	180000	0,00E+00	neg	neg
5546	Piedmont	WWTP7	17/10/2024		7,81E+05	1,92E-01	3,84E+03	3839940	7,80E+05	neg	neg
5475	Lazio	WWTP2	22/10/2024		1,71E+05	6,35E-01	1,27E+04	300000	7,22E+06	ME	neg
5476	Lazio	WWTP3	22/10/2024		7,43E+05	2,42E-01	4,84E+03	600000	6,00E+06	neg	neg
5477	Lazio	WWTP1	22/10/2024		1,70E+05	8,07E-02	1,61E+03	1100000	2,49E+05	neg	neg
5560	Sicily	WWTP8	22/10/2024		5,99E+04	1,44E-01	2,88E+03	880000	1,96E+05	neg	neg
5548	Piedmont	WWTP6	05/11/2024		2,63E+04	0,00E+00	0,00E+00	180000	0,00E+00	neg	neg

5490	Lazio	WWTP1	06/11/2024		1,04E+05	2,39E-01	4,78E+03	1100000	4,54E+05	G3	neg
5491	Lazio	WWTP2	06/11/2024		8,38E+05	1,62E-01	3,24E+03	300000	9,04E+06	G3	neg
5492	Lazio	WWTP3	06/11/2024		7,08E+05	7,21E-01	1,44E+04	600000	1,70E+07	G3	neg
5550	Piedmont	WWTP7	06/11/2024		6,42E+05	5,74E-01	1,15E+04	3839940	1,92E+06	neg	neg
5505	Lazio	WWTP5	07/11/2024		6,41E+04	2,66E-02	5,32E+02	350000	9,75E+04	neg	neg
5512	Lazio	WWTP1	20/11/2024		7,60E+05	4,33E-01	8,66E+03	1100000	5,98E+06	neg	neg
5513	Lazio	WWTP2	20/11/2024		1,07E+05	1,05E-01	2,11E+03	300000	7,50E+05	neg	neg
5514	Lazio	WWTP3	20/11/2024		1,69E+05	5,36E-01	1,07E+04	600000	3,02E+06	G3	neg
5561	Sicily	WWTP8	26/11/2024		5,73E+04	2,86E-01	5,73E+03	880000	3,73E+05	neg	neg
5552	Piedmont	WWTP6	03/12/2024		2,65E+04	7,17E-02	1,43E+03	180000	2,11E+05	neg	neg
5554	Piedmont	WWTP7	04/12/2024		5,64E+05	4,78E-02	9,57E+02	3839940	1,41E+05	neg	neg
5521	Lazio	WWTP2	17/12/2024		1,06E+05	1,23E+00	2,47E+04	300000	8,69E+06	G3	neg
5522	Lazio	WWTP3	17/12/2024		1,68E+05	1,78E+00	3,55E+04	600000	9,96E+06	G3	neg
5523	Lazio	WWTP1	17/12/2024		7,34E+05	1,05E+00	2,10E+04	1100000	1,41E+07	G3	neg
5562	Sicily	WWTP8	18/12/2024		6,04E+04	2,87E-01	5,74E+03	880000	3,93E+05	neg	neg
5527	Lazio	WWTP2	13/01/2025		1,08E+05	4,99E-01	9,98E+03	300000	3,60E+06	G2	neg
5528	Lazio	WWTP3	13/01/2025		1,72E+05	6,66E-01	1,33E+04	600000	3,83E+06	G3	neg
5529	Lazio	WWTP1	13/01/2025	Winter	6,91E+05	1,21E+00	2,42E+04	1100000	1,52E+07	neg	neg
5556	Piedmont	WWTP6	15/01/2025		2,46E+04	1,03E+00	2,06E+04	180000	2,81E+06	neg	neg
5558	Piedmont	WWTP7	15/01/2025		5,44E+05	4,06E-01	8,13E+03	3839940	1,15E+06	neg	neg
5530	Lazio	WWTP2	26/01/2025		1,01E+05	2,54E+00	5,08E+04	300000	1,72E+07	G2	neg
5531	Lazio	WWTP3	26/01/2025		1,73E+05	1,03E+00	2,06E+04	600000	5,93E+06	G9	neg
5532	Lazio	WWTP1	27/01/2025		7,08E+05	9,22E-01	1,84E+04	1100000	1,19E+07	G3	neg
5533	Lazio	WWTP1	28/01/2025		7,33E+05	0,00E+00	0,00E+00	1100000	0,00E+00	neg	neg
5563	Sicily	WWTP8	28/01/2025		5,80E+04	3,82E-01	7,65E+03	880000	5,04E+05	neg	neg
5541	Sicily	WWTP8	03/02/2025		6,02E+04	1,67E-01	3,35E+03	880000	2,29E+05	neg	neg
5539	Piedmont	WWTP7	04/02/2025		5,86E+05	2,39E-01	4,78E+03	3839940	7,29E+05	neg	neg

5602	Piedmont	WWTP7	05/02/2025		5,80E+05	1,23E+00	2,45E+04	3839940	3,70E+06	neg	neg
5612	Piedmont	WWTP6	05/02/2025		2,52E+04	5,26E-01	1,05E+04	180000	1,47E+06	neg	neg
5564	Lazio	WWTP1	10/02/2025		7,17E+05	6,72E-01	1,34E+04	1100000	8,77E+06	G3 (partial)	neg
5622	Sicily	WWTP8	17/02/2025		5,92E+04	5,26E-01	1,05E+04	880000	7,08E+05	neg	neg
5604	Piedmont	WWTP7	19/02/2025		5,60E+05	8,61E-01	1,72E+04	3839940	2,51E+06	neg	neg
5614	Piedmont	WWTP6	19/02/2025		2,49E+04	2,40E-01	4,79E+03	180000	6,62E+05	neg	neg
5566	Lazio	WWTP2	24/02/2025		1,09E+05	6,37E-01	1,27E+04	300000	4,62E+06	G9	neg
5567	Lazio	WWTP3	24/02/2025		1,66E+05	2,27E+00	4,54E+04	600000	1,26E+07	G9	neg
5568	Lazio	WWTP1	24/02/2025		7,69E+05	4,44E-01	8,87E+03	1100000	6,20E+06	G3	neg
5606	Piedmont	WWTP7	05/03/2025		6,02E+05	1,45E+00	2,90E+04	3839940	4,55E+06	neg	neg
5616	Piedmont	WWTP6	05/03/2025		2,56E+04	2,11E+00	4,21E+04	180000	5,99E+06	neg	neg
5569	Lazio	WWTP2	10/03/2025		1,05E+05	1,67E+00	3,33E+04	300000	1,17E+07	G3	neg
5570	Lazio	WWTP3	10/03/2025		1,61E+05	8,88E-01	1,78E+04	600000	4,76E+06	G9	neg
5571	Lazio	WWTP1	10/03/2025		7,30E+05	1,19E+00	2,38E+04	1100000	1,58E+07	G3	neg
5608	Piedmont	WWTP7	19/03/2025		6,20E+05	5,05E+00	1,01E+05	3839940	1,63E+07	G3	P8
5618	Piedmont	WWTP6	19/03/2025		2,32E+04	8,85E-01	1,77E+04	180000	2,28E+06	neg	neg
5572	Lazio	WWTP2	24/03/2025		1,29E+05	1,43E+00	2,87E+04	300000	1,23E+07	G3	neg
5573	Lazio	WWTP3	24/03/2025		1,80E+05	8,17E-01	1,63E+04	600000	4,90E+06	neg	neg
5574	Lazio	WWTP1	24/03/2025		7,27E+05	1,67E+00	3,34E+04	1100000	2,21E+07	G2	neg
5623	Sicily	WWTP8	25/03/2025		7,06E+04	4,79E-01	9,58E+03	880000	7,68E+05	neg	neg
5610	Piedmont	WWTP7	02/04/2025	Spring	5,25E+05	1,84E+00	3,68E+04	3839940	5,04E+06	neg	neg
5620	Piedmont	WWTP6	02/04/2025		2,66E+04	2,99E+00	5,98E+04	180000	8,84E+06	G3	neg
5591	Lazio	WWTP2	07/04/2025		1,09E+05	9,03E+00	1,81E+05	300000	6,54E+07	neg	neg
5592	Lazio	WWTP3	07/04/2025		1,67E+05	1,88E+01	3,75E+05	600000	1,04E+08	neg	neg
5593	Lazio	WWTP1	07/04/2025		7,14E+05	3,44E+00	6,88E+04	600000	8,18E+07	neg	neg
5624	Sicily	WWTP8	07/04/2025		6,16E+04	2,39E-02	4,79E+02	880000	3,35E+04	G3	neg

5594	Lazio	WWTP1	28/04/2025		7,15E+05	8,04E+00	1,61E+05	1100000	1,04E+08	neg	neg
5595	Lazio	WWTP2	28/04/2025		1,71E+05	8,22E+00	1,64E+05	300000	9,38E+07	G3	neg
5596	Lazio	WWTP3	28/04/2025		1,81E+05	6,70E+00	1,34E+05	600000	4,05E+07	neg	neg
5599	Sicily	WWTP8	05/05/2025		5,41E+04	2,37E+00	4,74E+04	880000	2,91E+06	neg	neg
5597	Piedmont	WWTP7	06/05/2025		6,42E+05	1,48E+00	2,97E+04	3839940	4,96E+06	neg	neg
5659	Piedmont	WWTP7	07/05/2025		7,32E+05	0,00E+00	0,00E+00	3839940	0,00E+00	neg	neg
5661	Piedmont	WWTP6	07/05/2025		4,28E+04	9,56E-02	1,91E+03	180000	4,55E+05	neg	neg
5625	Lazio	WWTP2	12/05/2025		1,04E+05	1,29E+01	2,58E+05	300000	8,93E+07	neg	neg
5626	Lazio	WWTP3	12/05/2025		1,82E+05	8,04E+00	1,61E+05	600000	4,87E+07	neg	neg
5627	Lazio	WWTP1	12/05/2025		6,86E+05	8,63E+00	1,73E+05	1100000	1,08E+08	neg	neg
5628	Lazio	WWTP2	26/05/2025		9,68E+04	4,02E+00	8,04E+04	300000	2,59E+07	neg	neg
5629	Lazio	WWTP3	26/05/2025		1,78E+05	7,65E+00	1,53E+05	600000	4,54E+07	neg	neg
5630	Lazio	WWTP1	26/05/2025		6,40E+05	1,94E-01	3,88E+03	1100000	2,26E+06	neg	neg
5631	Lazio	WWTP2	09/06/2025		9,47E+04	1,90E+01	3,80E+05	300000	1,20E+08	ME	neg
5632	Lazio	WWTP3	09/06/2025		1,27E+05	3,65E+01	7,30E+05	600000	1,54E+08	G3	neg
5663	Piedmont	WWTP7	11/06/2025		5,62E+05	0,00E+00	0,00E+00	3839940	0,00E+00	neg	neg
5665	Piedmont	WWTP6	11/06/2025		3,16E+04	0,00E+00	0,00E+00	180000	0,00E+00	neg	neg
5667	Piedmont	WWTP7	18/06/2025		5,29E+05	0,00E+00	0,00E+00	3839940	0,00E+00	neg	neg
5672	Sicily	WWTP8	23/06/2025	Summer	4,65E+04	1,20E-01	2,40E+03	180000	6,19E+05	G3	neg

ME= Mixed Electropherogram

Table S2. Rotavirus prototypes retrieved from the Rotavirus Genotyping Tool and employed in the construction of the phylogenetic tree

**Accession Number**	**Genotypes**

K02033	G1
D16320	G1
M92651	G1
DQ629928	G1
GQ433989	G1
AB118023	G2
KF501107	G2
AY261340	G2
FJ492832	G2
D86270	G3
KC254793	G3
AY707788	G3
KJ135167	G3
U97199	G3
AF528201	G3
AY750924	G3
AY707792	G3
AF386914	G3
M21650	G3
AB056650	G3
X13603	G4
M86833	G4
AB690413	G4
AJ488586	G4
X04613	G5
X65940	G6
D12710	G6
AF207063	G6

EF554109	G6
EF199501	G6
JX008866	G6
AB080737	G7
EF672560	G8
AB180969	G9
AY866500	G9
L14072	G9
FJ794173	G9
X63156	G10
M23194	G11
M58290	G12
D13549	G13
JF712582	G14
AB853895	G15
GQ479955	G16
S58166	G17
D82979	G18
JQ085407	G19
EU805775	G20
AB454421	G21
EU486973	G22
FN393054	G23
AB513837	G24
GU983676	G25

Table S3. Sequences Accession Number NCBI

ID Sample	Accession Number NCBI
5166	PX096785
5167	PX096786
5168	PX096787
5169	PX096788
5170	PX096789
5171	PX096790
5206	PX096791
5210	PX096792
5212	PX096793
5214	PX096794
5215	PX096795
5216	PX096796
5217	PX096797
5218	PX096798
5219	PX096780
5242	PX096799
5243	PX096800
5244	PX096801
5245	PX096802
5246	PX096803
5247	PX096804
5248	PX096805
5249	PX096806
5250	PX096807

5251	PX096808
5252	PX096809
5253	PX096810
5254	PX096781
5255	PX096811
5256	PX096812
5264	PX096813
5265	PX096814
5266	PX096817
5267	PX096815
5268	PX096782
5272	PX096816
5273	PX096818
	PX096819
5284	PX096820
5290	PX096821
5298	PX096822
5299	PX096783
5306	PX096774
5309	PX096823
5312	PX096773
5316	PX096825
5463	PX096776
5467	PX096775
5490	PX096824

5491	PX096826
5492	PX096827
5496	PX096828
5514	PX096829
5521	PX096831
5522	PX096830
5523	PX096832
5527	PX096772
5528	PX096833
5530	PX096784
5531	PX096834
5532	PX096835
5564	PX096836
5566	PX096837
5567	PX096838
5568	PX096839
5569	PX096840
5570	PX096841
5571	PX096842
5572	PX096843
5574	PX096844
5595	PX096778
5608	PX096845
5620	PX096777
5624	PX096846
5632	PX096779

5672	PX096847
